# Supplementary material for: Development of a bioassay-guided genome mining approach for antifungal natural product discovery from pseudomonads
Source: Sci Rep. 2026 May 2;16:15990. doi: 10.1038/s41598-026-48020-5 (PMC13197424; doi:10.1038/s41598-026-48020-5)
Supplement: Supplementary file 1 — Supplementary Material 1 [file 41598_2026_48020_MOESM1_ESM.docx]

**Supporting Information**


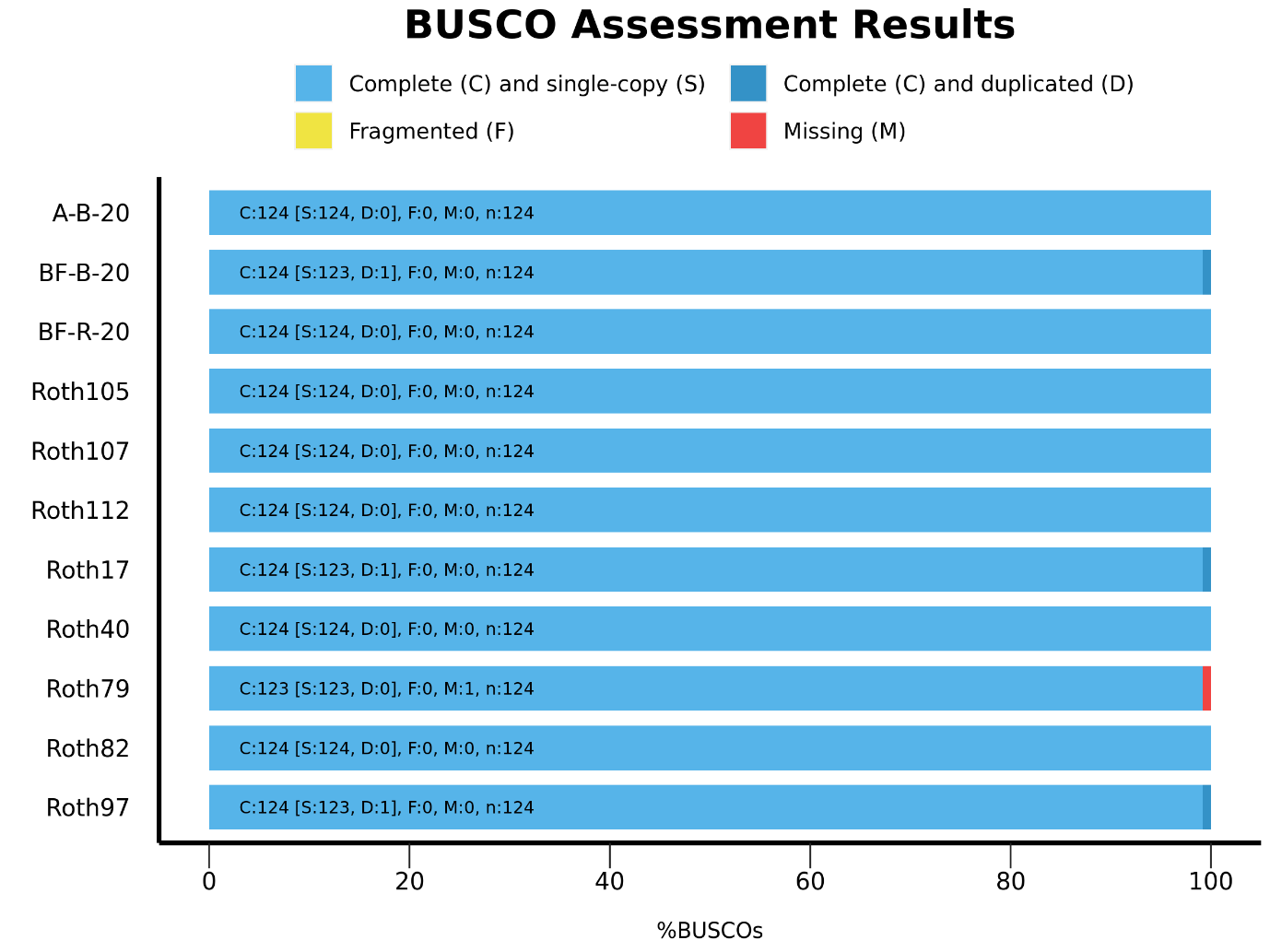


**Figure S1. A bar chart indicating the status of 124 BUSCO genes from the Bacteria odb10 dataset of the *Pseudomonas* genome assemblies utilised in this study.** All genome assemblies were found to show high completeness and low levels of contamination using the general bacterial dataset.


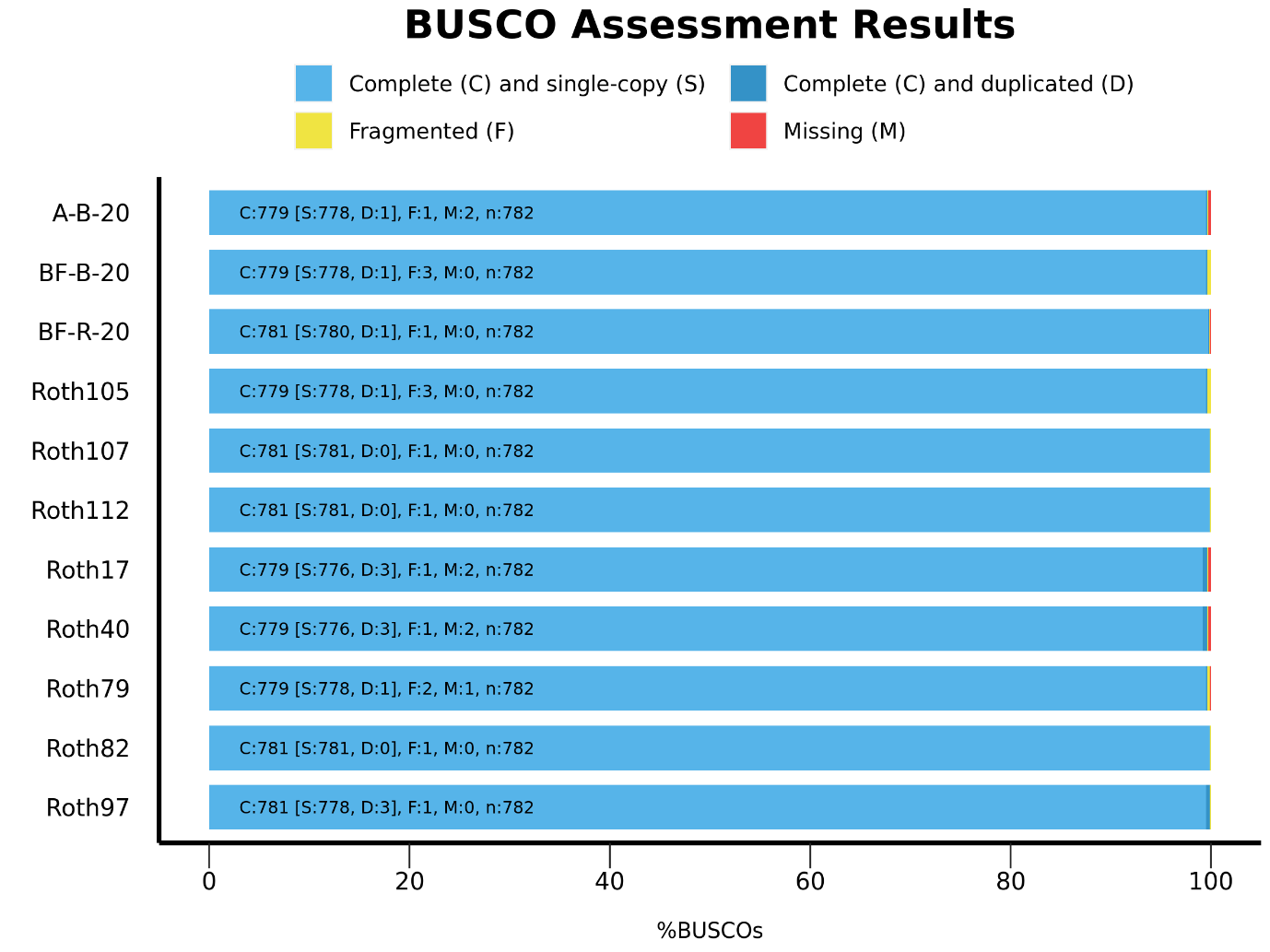


**Figure S2. A bar chart indicating the status of 782 BUSCO genes from the Pseudomonadales odb10 dataset of the *Pseudomonas* genome assemblies utilised in this study.** All genome assemblies were found to show high completeness and low levels of contamination using the Pseudomonadales dataset.


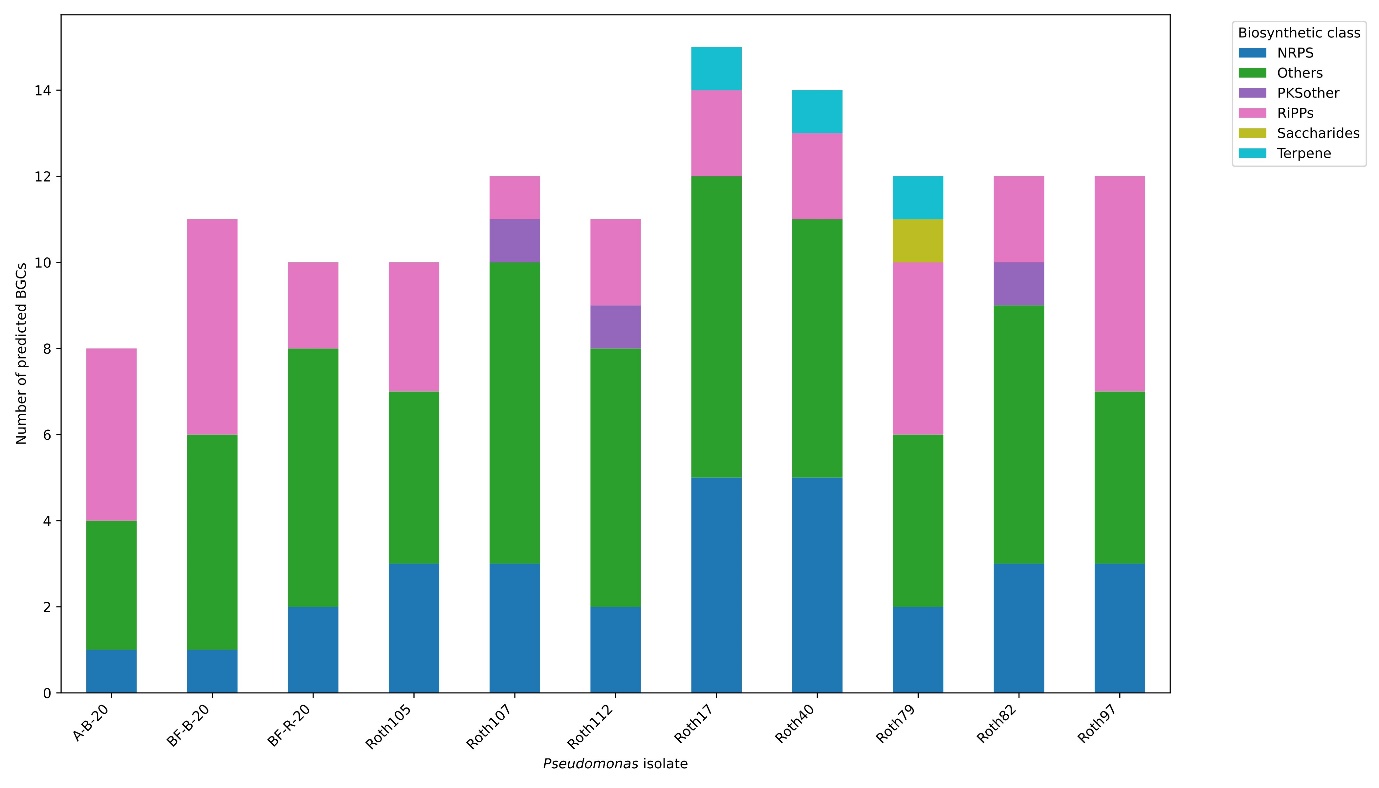


**Figure S3. Summary of predicted secondary metabolite biosynthetic gene cluster classes.** A bar chart summarising the number of predicted secondary metabolite biosynthetic gene clusters and their biosynthetic classes from the genome assemblies of each *Pseudomonas* strain tested within this study.

**
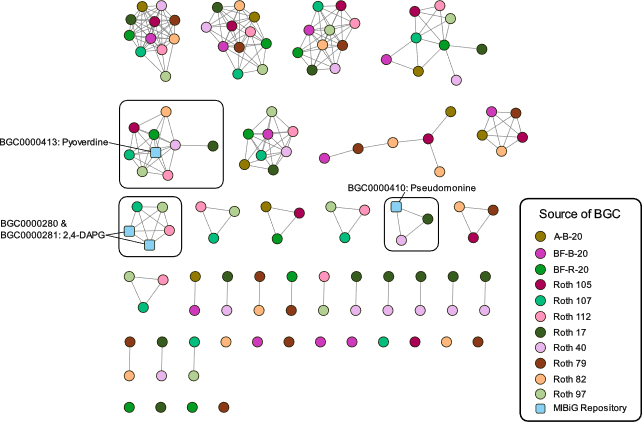
**

**Figure S4. BiG-SCAPE sequence similarity networks of predicted BGCs from the 11 *Pseudomonas* isolate genome assemblies.** Sequence similarity networks of predicted BGCs, with nodes coloured according to the isolate genome assembly from which they were predicted. Connected nodes indicate similarity at the 0.3 threshold.


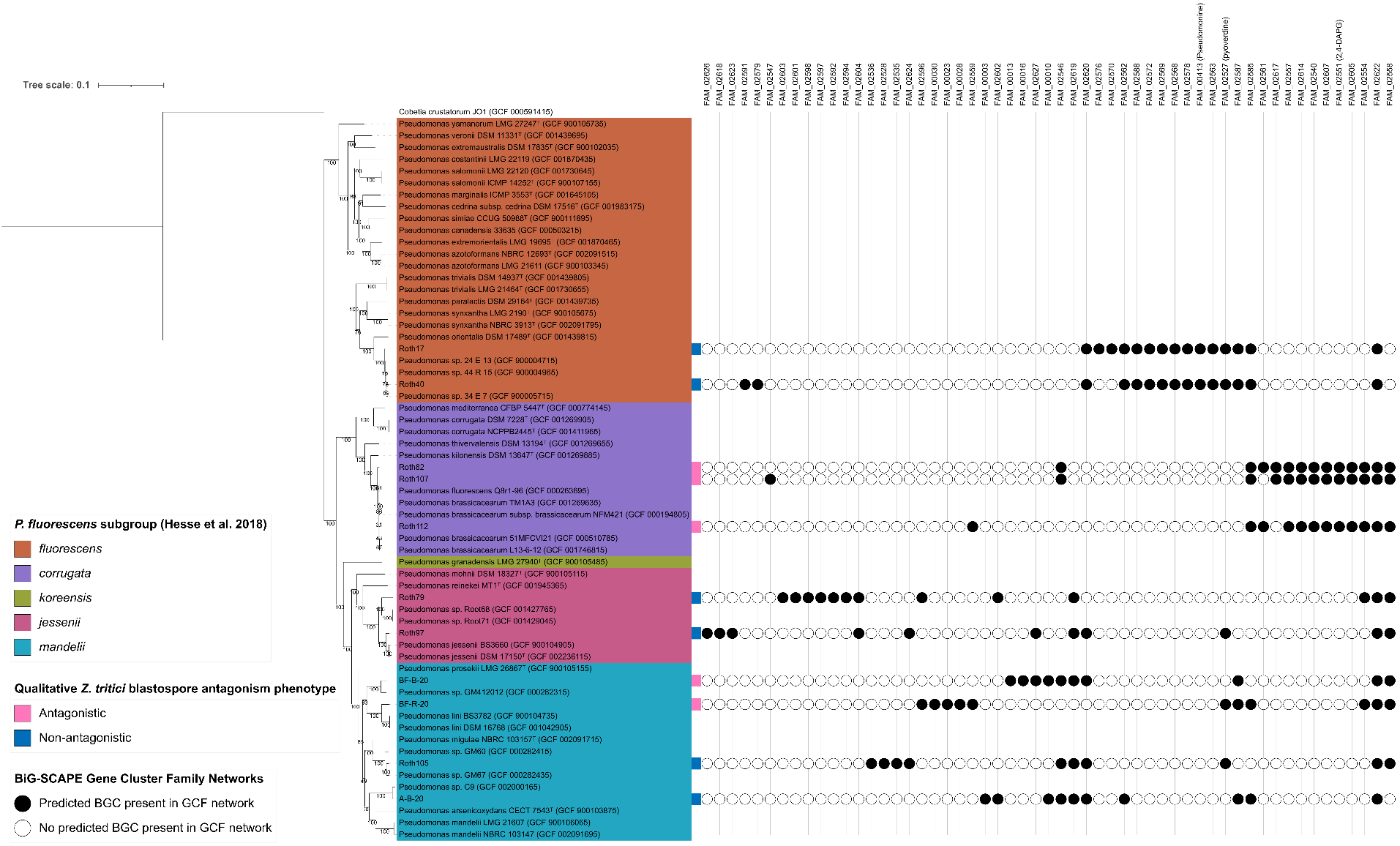


**Figure S5. Phylogenetic distribution of predicted secondary metabolite BGCs and *Z. tritici* blastopore antagonism phenotypes of *Pseudomonas* isolates used in this study.** Phylogenetic analysis with known *Pseudomonas* strains, using autoMLST, putatively identifies 11 *Pseudomonas* strains used within this study within the *P. fluorescens*, *P. corrugata*, *P. koreensis*, *P. jessenii*, and *P. mandelii* subgroups of the *P. fluorescens* species complex. The presence and absence of all predicted BGCs that form Gene Cluster Family sequence similarity networks (GCFs) are indicated, with those alongside qualitative *Z. tritici* blastospore antagonism phenotype against *Z. tritici* strain IPO323. Type strain *Pseudomonas* genomes identified according to LPSN (accessed 24^th^ January 2025) are denoted by a superscript ‘T’.


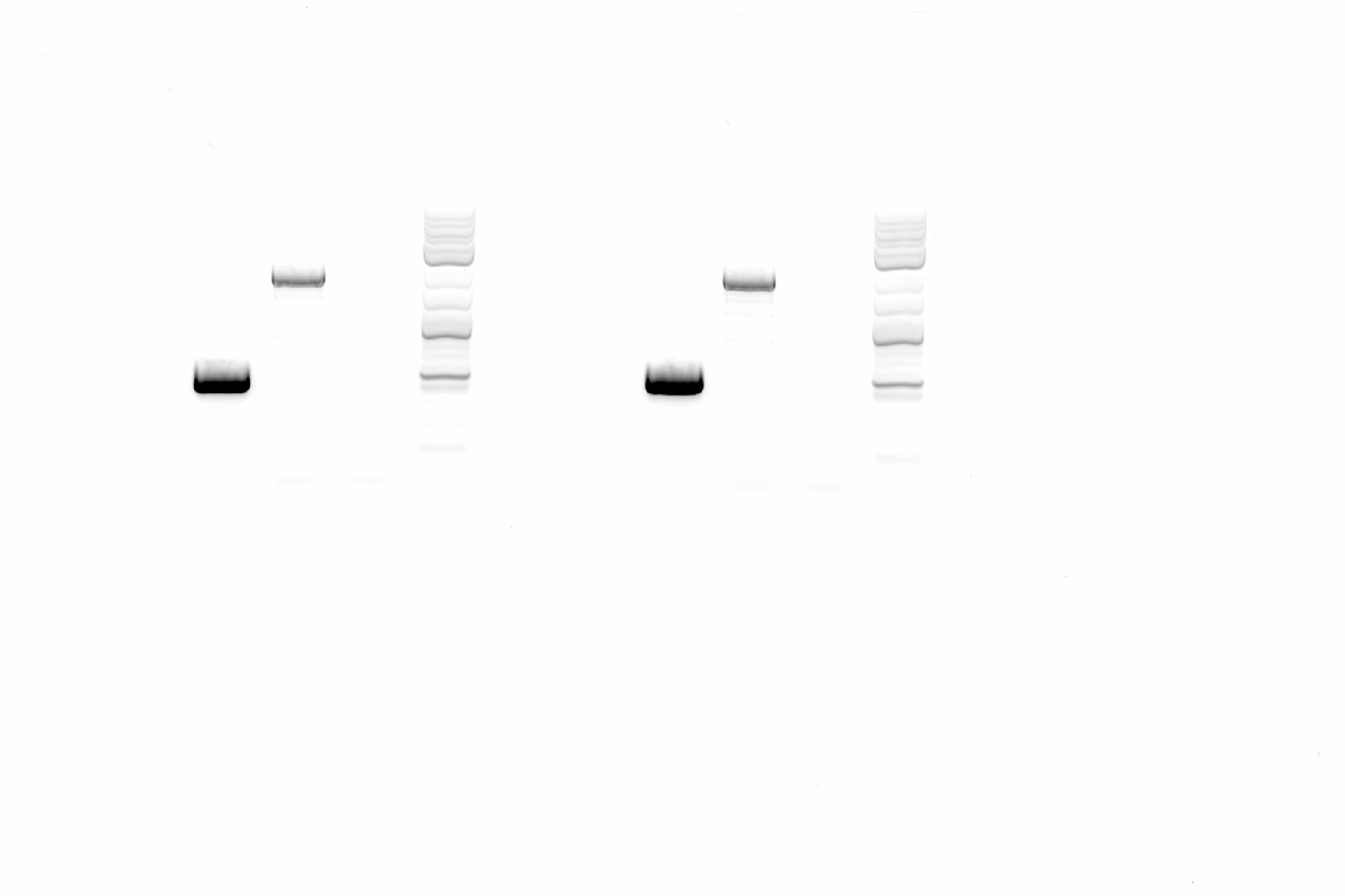


**Roth82**

**Roth82∆*phlD***

**water**


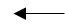

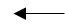


500bp

2000bp

3000bp

**Figure S6.** **Agarose gel electrophoresis of PCR (using external primers from the insertion region, pKnockPhlD2_Fw – pKnockPhlD2_Rv) for verification of *phlD* gene knockout.** Roth82 PCR product size is 527bp, Roth82*∆phlD* shows the insertion of pKnock-Km vector (2098bp).


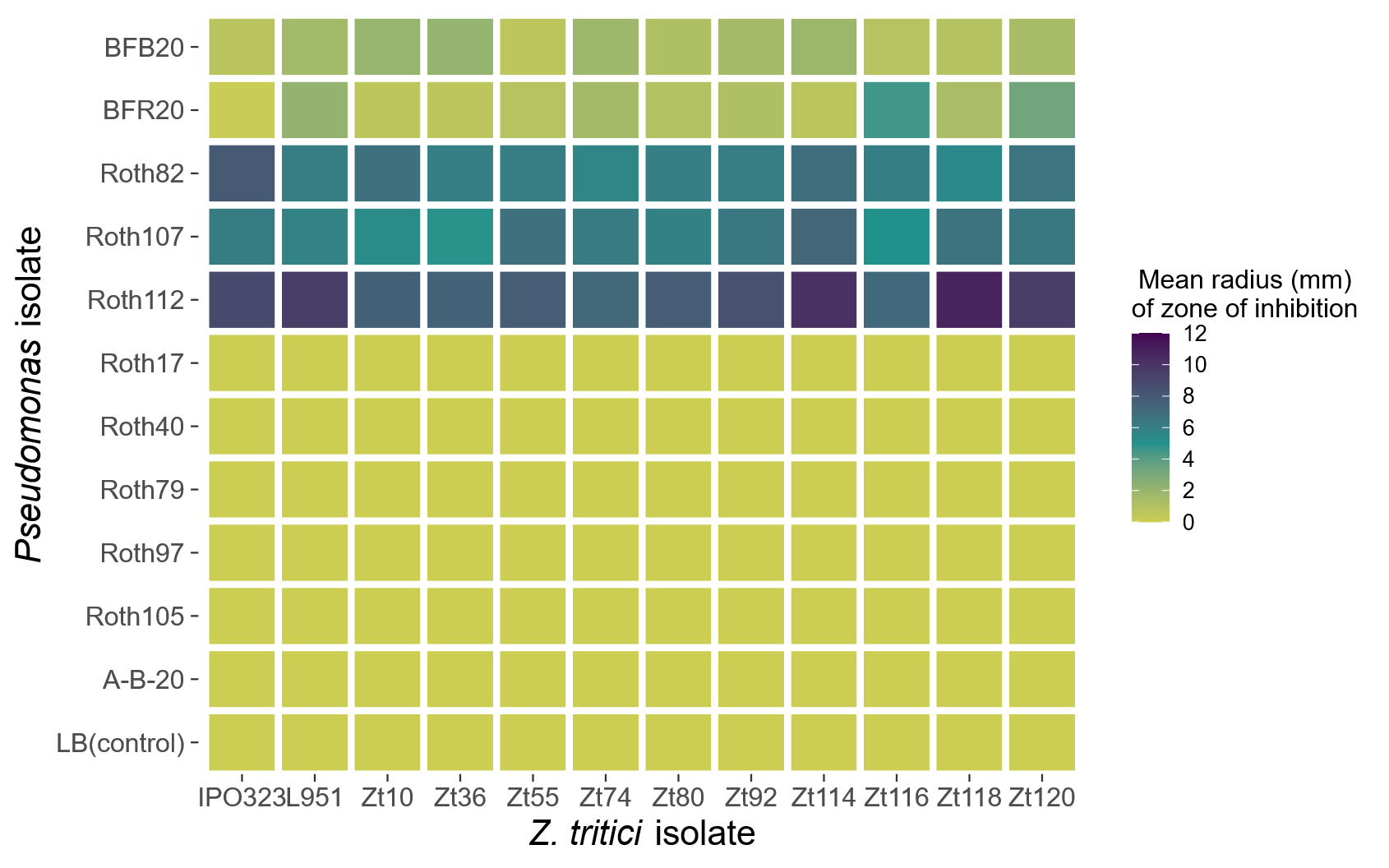


**Figure S7. Heatmap indicating the average radius of the zone of inhibition produced in each pairwise interaction between antagonistic *Pseudomonas* and *Z. tritici* isolates in the quantitative antagonism assay after 12 days (n=4), alongside LB media controls.**

**Table S1. 52 *Pseudomonas* isolates from the culture collection of 534 identified as antagonistic in qualitative antagonism assay against *Z. tritici* isolate IPO323.**

| ***Pseudomonas* isolate** | ***Z. tritici* qualitative blastospore antagonism phenotype** |
| --- | --- |
| 1E_1 | antagonistic |
| 1E_2 | antagonistic |
| 1R_3 | antagonistic |
| 22E_6 | antagonistic |
| 24E_10 | antagonistic |
| 24R_5 | antagonistic |
| 28E_14 | antagonistic |
| 28E_15 | antagonistic |
| 28R_11 | antagonistic |
| 28R_2 | antagonistic |
| 28R_7 | antagonistic |
| 30R_14 | antagonistic |
| 30R_16 | antagonistic |
| 30R_2 | antagonistic |
| 30R_8 | antagonistic |
| 31E_11 | antagonistic |
| 31R_14 | antagonistic |
| 35E_3 | antagonistic |
| 37R_16 | antagonistic |
| 37R_6 | antagonistic |
| 37R_7 | antagonistic |
| 37R_8 | antagonistic |
| 58E_12 | antagonistic |
| 58E_13 | antagonistic |
| 58R_1 | antagonistic |
| 58R_2 | antagonistic |
| 8E_1 | antagonistic |
| BF-B-20 | antagonistic |
| BF-R-20 | antagonistic |
| Roth103 | antagonistic |
| Roth104 | antagonistic |
| Roth107 | antagonistic |
| Roth111 | antagonistic |
| Roth112 | antagonistic |
| Roth12 | antagonistic |
| Roth16 | antagonistic |
| Roth20 | antagonistic |
| Roth21 | antagonistic |
| Roth26 | antagonistic |
| Roth4 | antagonistic |
| Roth40 | antagonistic |
| Roth54 | antagonistic |
| Roth56 | antagonistic |
| Roth58 | antagonistic |
| Roth64 | antagonistic |
| Roth65 | antagonistic |
| Roth70 | antagonistic |
| Roth73 | antagonistic |
| Roth79 | antagonistic |
| Roth81 | antagonistic |
| Roth82 | antagonistic |
| Roth83 | antagonistic |
| Roth97 | antagonistic |

**Table S2. Qualitative *Z. tritici* antagonism phenotype of *Pseudomonas* isolates used in the quantitative antagonism assay against 12 diverse *Z. tritici* isolates.**

| ***Pseudomonas* isolate** | ***Z. tritici* qualitative blastospore antagonism phenotype** |
| --- | --- |
| A-B-20 | non-antagonistic |
| BF-B-20 | antagonistic |
| BF-R-20 | antagonistic |
| Roth17 | non-antagonistic |
| Roth40 | non-antagonistic |
| Roth79 | non-antagonistic |
| Roth82 | antagonistic |
| Roth97 | non-antagonistic |
| Roth105 | non-antagonistic |
| Roth107 | antagonistic |
| Roth112 | antagonistic |

**Table S3. Genes utilised in the construction of the phylogenetic tree using autoMLST.**

| TIGR01067 |
| --- |
| TIGR01050 |
| TIGR01164 |
| TIGR01032 |
| TIGR01029 |
| TIGR00493 |
| TIGR00138 |
| TIGR01966 |
| TIGR03635 |
| TIGR03284 |
| TIGR00952 |
| TIGR02012 |
| TIGR01169 |
| TIGR01011 |
| TIGR00967 |
| TIGR00928 |
| TIGR03723 |
| TIGR00612 |
| TIGR01302 |
| TIGR00690 |
| TIGR00019 |
| TIGR00362 |
| TIGR01009 |
| TIGR00962 |
| TIGR00628 |
| TIGR00521 |
| TIGR01039 |
| TIGR03632 |
| TIGR00482 |
| TIGR01850 |
| TIGR00132 |
| TIGR01021 |
| TIGR00048 |
| TIGR00713 |
| TIGR00855 |
| TIGR02970 |
| TIGR00563 |
| TIGR02075 |
| TIGR01394 |
| TIGR01207 |
| TIGR00338 |
| TIGR00263 |
| TIGR00008 |
| TIGR00067 |
| TIGR02673 |
| TIGR00382 |
| TIGR02027 |
| TIGR00651 |
| TIGR00337 |
| TIGR00767 |
| TIGR00088 |
| TIGR00418 |
| TIGR03346 |
| TIGR01792 |
| TIGR00190 |
| TIGR00963 |
| TIGR01171 |
| TIGR00635 |
| TIGR00060 |
| TIGR00431 |
| TIGR00243 |
| TIGR02225 |
| TIGR00228 |
| TIGR00012 |
| TIGR00062 |
| TIGR02274 |
| TIGR03631 |
| TIGR01059 |
| TIGR00755 |
| TIGR01163 |
| TIGR00184 |
| TIGR00615 |
| TIGR00065 |
| TIGR00119 |
| TIGR00033 |
| TIGR00708 |
| TIGR01049 |
| TIGR00165 |
| TIGR01632 |
| TIGR03594 |
| TIGR00355 |
| TIGR00168 |
| TIGR00508 |
| TIGR00036 |
| TIGR01044 |
| TIGR00055 |
| TIGR00981 |
| TIGR01024 |
| TIGR01083 |
| TIGR00959 |
| TIGR00430 |
| TIGR00118 |
| TIGR00459 |
| TIGR02729 |
| TIGR00499 |
| TIGR01816 |
| TIGR02013 |
| TIGR00580 |
| TIGR02386 |
|  |

Table S4. One-way Analysis of Variance of the radius of zones of inhibition produced by *Pseudomonas* isolate BFR20 in confrontation with genetically diverse *Z. tritici* isolates in the blastospore antagonism assay *in vitro*.

Measurements were log transformed to achieve approximate normality.

|  | Df | Sum Sq | Mean Sq | *F* value | Pr(>*F*) |  |
| --- | --- | --- | --- | --- | --- | --- |
| *Z. tritici* genotype | 11 | 45.198 | 4.109 | 11.476 | 9.423E-09 | *** |
| Residuals | 36 | 12.889 | 0.358 |  |  |  |

Significance codes: 0 ‘***’ 0.001 ‘**’ 0.01 ‘*’ 0.05 ‘.’ 0.1 ‘ ’ 1

Table S5. Tukey’s test for multiple comparisons at the 5% level of significance, comparing zones of inhibition produced by *Pseudomonas* isolate BFR20 in confrontation with different *Z. tritici* genotypes *in vitro*.

Measurements were log transformed to achieve approximate normality.

| Comparison | Mean Difference | Lower Bound | Upper Bound | Padj |
| --- | --- | --- | --- | --- |
| L951-IPO323 | 3.3892755 | 1.91250399 | 4.86604702 | 0.0000001 |
| Zt10-IPO323 | 2.1189732 | 0.64220166 | 3.59574469 | 0.0007863 |
| Zt114-IPO323 | 2.3035831 | 0.82681158 | 3.78035461 | 0.0002151 |
| Zt116-IPO323 | 3.6951467 | 2.21837519 | 5.17191822 | 0 |
| Zt118-IPO323 | 2.9357476 | 1.45897613 | 4.41251916 | 0.0000024 |
| Zt120-IPO323 | 3.8555348 | 2.37876325 | 5.33230628 | 0 |
| Zt36-IPO323 | 1.9692034 | 0.49243186 | 3.44597489 | 0.0022042 |
| Zt55-IPO323 | 2.5081041 | 1.0313326 | 3.98487563 | 0.0000502 |
| Zt74-IPO323 | 3.0945381 | 1.61776662 | 4.57130965 | 0.0000008 |
| Zt80-IPO323 | 2.7117675 | 1.23499602 | 4.18853905 | 0.0000118 |
| Zt92-IPO323 | 2.8210343 | 1.34426279 | 4.29780583 | 0.0000054 |
| Zt10-L951 | -1.2703023 | -2.74707384 | 0.20646919 | 0.1494898 |
| Zt114-L951 | -1.0856924 | -2.56246393 | 0.39107911 | 0.3348054 |
| Zt116-L951 | 0.3058712 | -1.17090032 | 1.78264272 | 0.9998325 |
| Zt118-L951 | -0.4535279 | -1.93029938 | 1.02324365 | 0.9941461 |
| Zt120-L951 | 0.4662593 | -1.01051225 | 1.94303078 | 0.9926561 |
| Zt36-L951 | -1.4200721 | -2.89684365 | 0.05669938 | 0.0686984 |
| Zt55-L951 | -0.8811714 | -2.35794291 | 0.59560013 | 0.6372617 |
| Zt74-L951 | -0.2947374 | -1.77150888 | 1.18203415 | 0.999883 |
| Zt80-L951 | -0.677508 | -2.15427948 | 0.79926355 | 0.8974541 |
| Zt92-L951 | -0.5682412 | -2.04501271 | 0.90853032 | 0.9669885 |
| Zt114-Zt10 | 0.1846099 | -1.2921616 | 1.66138143 | 0.999999 |
| Zt116-Zt10 | 1.5761735 | 0.09940201 | 3.05294504 | 0.027913 |
| Zt118-Zt10 | 0.8167745 | -0.65999705 | 2.29354598 | 0.7334086 |
| Zt120-Zt10 | 1.7365616 | 0.25979008 | 3.21333311 | 0.0102865 |
| Zt36-Zt10 | -0.1497698 | -1.62654132 | 1.32700171 | 0.9999999 |
| Zt55-Zt10 | 0.3891309 | -1.08764058 | 1.86590245 | 0.9984292 |
| Zt74-Zt10 | 0.975565 | -0.50120655 | 2.45233648 | 0.4909093 |
| Zt80-Zt10 | 0.5927944 | -0.88397715 | 2.06956588 | 0.9558116 |
| Zt92-Zt10 | 0.7020611 | -0.77471038 | 2.17883265 | 0.8744014 |
| Zt116-Zt114 | 1.3915636 | -0.08520791 | 2.86833513 | 0.080234 |
| Zt118-Zt114 | 0.6321645 | -0.84460697 | 2.10893606 | 0.9326965 |
| Zt120-Zt114 | 1.5519517 | 0.07518016 | 3.02872319 | 0.032264 |
| Zt36-Zt114 | -0.3343797 | -1.81115124 | 1.14239179 | 0.9996084 |
| Zt55-Zt114 | 0.204521 | -1.2722505 | 1.68129254 | 0.9999971 |
| Zt74-Zt114 | 0.790955 | -0.68581647 | 2.26772656 | 0.7692799 |
| Zt80-Zt114 | 0.4081844 | -1.06858707 | 1.88495596 | 0.9976067 |
| Zt92-Zt114 | 0.5174512 | -0.9593203 | 1.99422273 | 0.9833483 |
| Zt118-Zt116 | -0.7593991 | -2.23617058 | 0.71737245 | 0.8101745 |
| Zt120-Zt116 | 0.1603881 | -1.31638345 | 1.63715958 | 0.9999998 |
| Zt36-Zt116 | -1.7259433 | -3.20271485 | -0.24917182 | 0.0110099 |
| Zt55-Zt116 | -1.1870426 | -2.66381411 | 0.28972893 | 0.2201155 |
| Zt74-Zt116 | -0.6006086 | -2.07738008 | 0.87616295 | 0.9517499 |
| Zt80-Zt116 | -0.9833792 | -2.46015068 | 0.49339235 | 0.47905 |
| Zt92-Zt116 | -0.8741124 | -2.35088391 | 0.60265912 | 0.6481235 |
| Zt120-Zt118 | 0.9197871 | -0.55698439 | 2.39655864 | 0.5772292 |
| Zt36-Zt118 | -0.9665443 | -2.44331579 | 0.51022725 | 0.5046965 |
| Zt55-Zt118 | -0.4276435 | -1.90441504 | 1.04912799 | 0.9964248 |
| Zt74-Zt118 | 0.1587905 | -1.31798102 | 1.63556201 | 0.9999998 |
| Zt80-Zt118 | -0.2239801 | -1.70075162 | 1.25279141 | 0.9999925 |
| Zt92-Zt118 | -0.1147133 | -1.59148485 | 1.36205818 | 1 |
| Zt36-Zt120 | -1.8863314 | -3.36310291 | -0.40955988 | 0.0038541 |
| Zt55-Zt120 | -1.3474307 | -2.82420217 | 0.12934086 | 0.1013749 |
| Zt74-Zt120 | -0.7609966 | -2.23776815 | 0.71577488 | 0.8081911 |
| Zt80-Zt120 | -1.1437672 | -2.62053875 | 0.33300428 | 0.2652239 |
| Zt92-Zt120 | -1.0345005 | -2.51127197 | 0.44227106 | 0.4040227 |
| Zt55-Zt36 | 0.5389007 | -0.93787077 | 2.01567226 | 0.9774503 |
| Zt74-Zt36 | 1.1253348 | -0.35143675 | 2.60210628 | 0.2862063 |
| Zt80-Zt36 | 0.7425642 | -0.73420735 | 2.21933568 | 0.8304654 |
| Zt92-Zt36 | 0.8518309 | -0.62494058 | 2.32860245 | 0.681987 |
| Zt74-Zt55 | 0.586434 | -0.89033749 | 2.06320554 | 0.9589337 |
| Zt80-Zt55 | 0.2036634 | -1.27310809 | 1.68043494 | 0.9999972 |
| Zt92-Zt55 | 0.3129302 | -1.16384132 | 1.78970171 | 0.9997914 |
| Zt80-Zt74 | -0.3827706 | -1.85954212 | 1.09400091 | 0.9986441 |
| Zt92-Zt74 | -0.2735038 | -1.75027534 | 1.20326769 | 0.9999439 |
| Zt92-Zt80 | 0.1092668 | -1.36750474 | 1.58603829 | 1 |

Table S6. One-way Analysis of Variance of the radius of zones of inhibition produced by *Pseudomonas* isolate BFB20 in confrontation with genetically diverse *Z. tritici* isolates in the blastospore antagonism assay *in vitro*.

|  | Df | Sum Sq | Mean Sq | *F* value | Pr(>*F*) |  |
| --- | --- | --- | --- | --- | --- | --- |
| *Z. tritici* genotype | 11 | 11.846 | 1.0769 | 10.97 | 1.70E-08 | *** |
| Residuals | 36 | 3.535 | 0.0982 |  |  |  |

Significance codes: 0 ‘***’ 0.001 ‘**’ 0.01 ‘*’ 0.05 ‘.’ 0.1 ‘ ’ 1

Table S7. Tukey’s test for multiple comparisons at the 5% level of significance, comparing zones of inhibition produced by *Pseudomonas* isolate BFB20 in confrontation with different *Z. tritici* genotypes *in vitro*.

| Comparison | Mean Difference | Lower Bound | Upper Bound | Padj |
| --- | --- | --- | --- | --- |
| L951-IPO323 | 0.87613032 | 0.10274959 | 1.649511048 | 0.0153963 |
| Zt10-IPO323 | 1.27820922 | 0.50482849 | 2.051589949 | 0.0000812 |
| Zt114-IPO323 | 1.11975177 | 0.34637104 | 1.893132502 | 0.0006879 |
| Zt116-IPO323 | 0.12457004 | -0.64881069 | 0.897950764 | 0.9999862 |
| Zt118-IPO323 | 0.17792996 | -0.59545076 | 0.951310693 | 0.9995452 |
| Zt120-IPO323 | 0.67016844 | -0.10321229 | 1.443549169 | 0.1427984 |
| Zt36-IPO323 | 1.36719858 | 0.59381785 | 2.140579311 | 0.0000242 |
| Zt55-IPO323 | -0.10867021 | -0.88205094 | 0.664710516 | 0.9999966 |
| Zt74-IPO323 | 1.08843085 | 0.31505012 | 1.86181158 | 0.0010422 |
| Zt80-IPO323 | 0.40976064 | -0.36362009 | 1.183141367 | 0.78066 |
| Zt92-IPO323 | 0.83261525 | 0.05923452 | 1.605995977 | 0.0256969 |
| Zt10-L951 | 0.4020789 | -0.37130183 | 1.175459629 | 0.7996747 |
| Zt114-L951 | 0.24362145 | -0.52975927 | 1.017002183 | 0.9927912 |
| Zt116-L951 | -0.75156028 | -1.52494101 | 0.021820445 | 0.0632204 |
| Zt118-L951 | -0.69820035 | -1.47158108 | 0.075180374 | 0.1091171 |
| Zt120-L951 | -0.20596188 | -0.97934261 | 0.56741885 | 0.9982739 |
| Zt36-L951 | 0.49106826 | -0.28231247 | 1.264448991 | 0.5493102 |
| Zt55-L951 | -0.98480053 | -1.75818126 | -0.211419803 | 0.0040078 |
| Zt74-L951 | 0.21230053 | -0.5610802 | 0.985681261 | 0.9977461 |
| Zt80-L951 | -0.46636968 | -1.23975041 | 0.307011048 | 0.6227831 |
| Zt92-L951 | -0.04351507 | -0.8168958 | 0.729865658 | 1 |
| Zt114-Zt10 | -0.15845745 | -0.93183818 | 0.614923282 | 0.9998491 |
| Zt116-Zt10 | -1.15363918 | -1.92701991 | -0.380258455 | 0.0004374 |
| Zt118-Zt10 | -1.10027926 | -1.87365998 | -0.326898526 | 0.000891 |
| Zt120-Zt10 | -0.60804078 | -1.38142151 | 0.165339949 | 0.2465025 |
| Zt36-Zt10 | 0.08898936 | -0.68439137 | 0.862370091 | 0.9999996 |
| Zt55-Zt10 | -1.38687943 | -2.16026016 | -0.613498704 | 0.0000185 |
| Zt74-Zt10 | -0.18977837 | -0.9631591 | 0.58360236 | 0.9991741 |
| Zt80-Zt10 | -0.86844858 | -1.64182931 | -0.095067853 | 0.0168749 |
| Zt92-Zt10 | -0.44559397 | -1.2189747 | 0.327786757 | 0.6834417 |
| Zt116-Zt114 | -0.99518174 | -1.76856247 | -0.221801009 | 0.0035101 |
| Zt118-Zt114 | -0.94182181 | -1.71520254 | -0.16844108 | 0.0068922 |
| Zt120-Zt114 | -0.44958333 | -1.22296406 | 0.323797396 | 0.6719591 |
| Zt36-Zt114 | 0.24744681 | -0.52593392 | 1.020827537 | 0.991822 |
| Zt55-Zt114 | -1.22842199 | -2.00180271 | -0.455041257 | 0.0001596 |
| Zt74-Zt114 | -0.03132092 | -0.80470165 | 0.742059807 | 1 |
| Zt80-Zt114 | -0.70999114 | -1.48337186 | 0.063389594 | 0.0970733 |
| Zt92-Zt114 | -0.28713653 | -1.06051725 | 0.486244204 | 0.9744221 |
| Zt118-Zt116 | 0.05335993 | -0.7200208 | 0.826740658 | 1 |
| Zt120-Zt116 | 0.5455984 | -0.22778232 | 1.318979133 | 0.3937203 |
| Zt36-Zt116 | 1.24262855 | 0.46924782 | 2.016009275 | 0.0001317 |
| Zt55-Zt116 | -0.23324025 | -1.00662098 | 0.540140481 | 0.9949645 |
| Zt74-Zt116 | 0.96386082 | 0.19048009 | 1.737241545 | 0.0052268 |
| Zt80-Zt116 | 0.2851906 | -0.48819013 | 1.058571332 | 0.9756552 |
| Zt92-Zt116 | 0.70804521 | -0.06533552 | 1.481425942 | 0.0989799 |
| Zt120-Zt118 | 0.49223848 | -0.28114225 | 1.265619204 | 0.545834 |
| Zt36-Zt118 | 1.18926862 | 0.41588789 | 1.962649346 | 0.0002709 |
| Zt55-Zt118 | -0.28660018 | -1.05998091 | 0.486780552 | 0.9747666 |
| Zt74-Zt118 | 0.91050089 | 0.13712016 | 1.683881616 | 0.0101527 |
| Zt80-Zt118 | 0.23183067 | -0.54155006 | 1.005211403 | 0.9952131 |
| Zt92-Zt118 | 0.65468528 | -0.11869545 | 1.428066013 | 0.1647201 |
| Zt36-Zt120 | 0.69703014 | -0.07635059 | 1.470410871 | 0.1103778 |
| Zt55-Zt120 | -0.77883865 | -1.55221938 | -0.005457924 | 0.0471057 |
| Zt74-Zt120 | 0.41826241 | -0.35511832 | 1.19164314 | 0.7587653 |
| Zt80-Zt120 | -0.2604078 | -1.03378853 | 0.512972927 | 0.9877408 |
| Zt92-Zt120 | 0.16244681 | -0.61093392 | 0.935827537 | 0.9998082 |
| Zt55-Zt36 | -1.47586879 | -2.24924952 | -0.702488066 | 0.0000055 |
| Zt74-Zt36 | -0.27876773 | -1.05214846 | 0.494612998 | 0.9794097 |
| Zt80-Zt36 | -0.95743794 | -1.73081867 | -0.184057215 | 0.0056674 |
| Zt92-Zt36 | -0.53458333 | -1.30796406 | 0.238797395 | 0.4236466 |
| Zt74-Zt55 | 1.19710106 | 0.42372034 | 1.970481793 | 0.0002438 |
| Zt80-Zt55 | 0.51843085 | -0.25494988 | 1.29181158 | 0.4691538 |
| Zt92-Zt55 | 0.94128546 | 0.16790473 | 1.71466619 | 0.0069385 |
| Zt80-Zt74 | -0.67867021 | -1.45205094 | 0.094710516 | 0.1317931 |
| Zt92-Zt74 | -0.2558156 | -1.02919633 | 0.517565126 | 0.9893376 |
| Zt92-Zt80 | 0.42285461 | -0.35052612 | 1.196235339 | 0.7465985 |

Table S8. One-way Analysis of Variance of the radius of zones of inhibition produced by *Pseudomonas* isolate Roth82 in confrontation with genetically diverse *Z. tritici* isolates in the blastospore antagonism assay *in vitro*.

|  | Df | Sum Sq | Mean Sq | *F* value | Pr(>*F*) |  |
| --- | --- | --- | --- | --- | --- | --- |
| *Z. tritici* genotype | 11 | 20.65 | 1.8769 | 4.455 | 3.09E-04 | *** |
| Residuals | 36 | 15.16 | 0.4213 |  |  |  |

Significance codes: 0 ‘***’ 0.001 ‘**’ 0.01 ‘*’ 0.05 ‘.’ 0.1 ‘ ’ 1

Table S9. Tukey’s test for multiple comparisons at the 5% level of significance, comparing zones of inhibition produced by *Pseudomonas* isolate Roth82 in confrontation with different *Z. tritici* genotypes *in vitro*.

| Comparison | Mean Difference | Lower Bound | Upper Bound | Padj |
| --- | --- | --- | --- | --- |
| L951-IPO323 | -1.930474289 | -3.5323385 | -0.3286101 | 0.0077849 |
| Zt10-IPO323 | -1.235944148 | -2.8378084 | 0.3659201 | 0.2700704 |
| Zt114-IPO323 | -1.158492905 | -2.7603571 | 0.4433713 | 0.357921 |
| Zt116-IPO323 | -2.043147161 | -3.6450114 | -0.441283 | 0.0039252 |
| Zt118-IPO323 | -2.58317819 | -4.1850424 | -0.981314 | 0.0001238 |
| Zt120-IPO323 | -1.4358289 | -3.0376931 | 0.1660353 | 0.1145686 |
| Zt36-IPO323 | -2.016143615 | -3.6180078 | -0.4142794 | 0.004633 |
| Zt55-IPO323 | -1.930917553 | -3.5327818 | -0.3290533 | 0.0077643 |
| Zt74-IPO323 | -2.34201241 | -3.9438766 | -0.7401482 | 0.0005943 |
| Zt80-IPO323 | -2.011981383 | -3.6138456 | -0.4101172 | 0.0047525 |
| Zt92-IPO323 | -1.993377658 | -3.5952419 | -0.3915135 | 0.0053238 |
| Zt10-L951 | 0.694530141 | -0.9073341 | 2.2963943 | 0.927074 |
| Zt114-L951 | 0.771981383 | -0.8298828 | 2.3738456 | 0.8646035 |
| Zt116-L951 | -0.112672873 | -1.7145371 | 1.4891913 | 1 |
| Zt118-L951 | -0.652703902 | -2.2545681 | 0.9491603 | 0.9511446 |
| Zt120-L951 | 0.494645388 | -1.1072188 | 2.0965096 | 0.9938759 |
| Zt36-L951 | -0.085669326 | -1.6875335 | 1.5161949 | 1 |
| Zt55-L951 | -0.000443265 | -1.6023075 | 1.6014209 | 1 |
| Zt74-L951 | -0.411538121 | -2.0134023 | 1.1903261 | 0.9987476 |
| Zt80-L951 | -0.081507095 | -1.6833713 | 1.5203571 | 1 |
| Zt92-L951 | -0.062903369 | -1.6647676 | 1.5389608 | 1 |
| Zt114-Zt10 | 0.077451243 | -1.524413 | 1.6793154 | 1 |
| Zt116-Zt10 | -0.807203013 | -2.4090672 | 0.7946612 | 0.8285826 |
| Zt118-Zt10 | -1.347234042 | -2.9490982 | 0.2546302 | 0.1711614 |
| Zt120-Zt10 | -0.199884753 | -1.801749 | 1.4019795 | 0.999999 |
| Zt36-Zt10 | -0.780199467 | -2.3820637 | 0.8216647 | 0.8566073 |
| Zt55-Zt10 | -0.694973405 | -2.2968376 | 0.9068908 | 0.9267834 |
| Zt74-Zt10 | -1.106068262 | -2.7079325 | 0.4957959 | 0.4252301 |
| Zt80-Zt10 | -0.776037235 | -2.3779014 | 0.825827 | 0.860689 |
| Zt92-Zt10 | -0.75743351 | -2.3592977 | 0.8444307 | 0.8781279 |
| Zt116-Zt114 | -0.884654256 | -2.4865185 | 0.71721 | 0.7351242 |
| Zt118-Zt114 | -1.424685285 | -3.0265495 | 0.1771789 | 0.1207081 |
| Zt120-Zt114 | -0.277335995 | -1.8792002 | 1.3245282 | 0.9999713 |
| Zt36-Zt114 | -0.857650709 | -2.4595149 | 0.7442135 | 0.7696577 |
| Zt55-Zt114 | -0.772424648 | -2.3742889 | 0.8294396 | 0.8641787 |
| Zt74-Zt114 | -1.183519504 | -2.7853837 | 0.4183447 | 0.3279098 |
| Zt80-Zt114 | -0.853488478 | -2.4553527 | 0.7483757 | 0.7748113 |
| Zt92-Zt114 | -0.834884753 | -2.436749 | 0.7669795 | 0.7972338 |
| Zt118-Zt116 | -0.540031029 | -2.1418952 | 1.0618332 | 0.9876231 |
| Zt120-Zt116 | 0.607318261 | -0.9945459 | 2.2091825 | 0.9702701 |
| Zt36-Zt116 | 0.027003547 | -1.5748607 | 1.6288678 | 1 |
| Zt55-Zt116 | 0.112229608 | -1.4896346 | 1.7140938 | 1 |
| Zt74-Zt116 | -0.298865249 | -1.9007295 | 1.302999 | 0.9999396 |
| Zt80-Zt116 | 0.031165778 | -1.5706984 | 1.63303 | 1 |
| Zt92-Zt116 | 0.049769503 | -1.5520947 | 1.6516337 | 1 |
| Zt120-Zt118 | 1.14734929 | -0.4545149 | 2.7492135 | 0.3717485 |
| Zt36-Zt118 | 0.567034576 | -1.0348296 | 2.1688988 | 0.9820156 |
| Zt55-Zt118 | 0.652260637 | -0.9496036 | 2.2541248 | 0.9513652 |
| Zt74-Zt118 | 0.241165781 | -1.3606984 | 1.84303 | 0.9999931 |
| Zt80-Zt118 | 0.571196807 | -1.0306674 | 2.173061 | 0.9810012 |
| Zt92-Zt118 | 0.589800532 | -1.0120637 | 2.1916647 | 0.9759244 |
| Zt36-Zt120 | -0.580314714 | -2.1821789 | 1.0215495 | 0.9786267 |
| Zt55-Zt120 | -0.495088653 | -2.0969529 | 1.1067756 | 0.9938306 |
| Zt74-Zt120 | -0.906183509 | -2.5080477 | 0.6956807 | 0.7063957 |
| Zt80-Zt120 | -0.576152483 | -2.1780167 | 1.0257117 | 0.9797371 |
| Zt92-Zt120 | -0.557548758 | -2.159413 | 1.0443154 | 0.9841718 |
| Zt55-Zt36 | 0.085226062 | -1.5166381 | 1.6870903 | 1 |
| Zt74-Zt36 | -0.325868795 | -1.927733 | 1.2759954 | 0.9998592 |
| Zt80-Zt36 | 0.004162232 | -1.597702 | 1.6060264 | 1 |
| Zt92-Zt36 | 0.022765957 | -1.5790983 | 1.6246302 | 1 |
| Zt74-Zt55 | -0.411094857 | -2.0129591 | 1.1907694 | 0.9987597 |
| Zt80-Zt55 | -0.08106383 | -1.682928 | 1.5208004 | 1 |
| Zt92-Zt55 | -0.062460105 | -1.6643243 | 1.5394041 | 1 |
| Zt80-Zt74 | 0.330031027 | -1.2718332 | 1.9318952 | 0.9998408 |
| Zt92-Zt74 | 0.348634752 | -1.2532295 | 1.950499 | 0.9997307 |
| Zt92-Zt80 | 0.018603725 | -1.5832605 | 1.6204679 | 1 |

Table S10. One-way Analysis of Variance of the radius of zones of inhibition produced by *Pseudomonas* isolate Roth107 in confrontation with genetically diverse *Z. tritici* isolates in the blastospore antagonism assay *in vitro*.

|  | Df | Sum Sq | Mean Sq | *F* value | Pr(>*F*) |  |
| --- | --- | --- | --- | --- | --- | --- |
| *Z. tritici* genotype | 11 | 23.32 | 2.1203 | 5.567 | 3.94E-05 | *** |
| Residuals | 36 | 13.71 | 0.3809 |  |  |  |

Significance codes: 0 ‘***’ 0.001 ‘**’ 0.01 ‘*’ 0.05 ‘.’ 0.1 ‘ ’ 1

Table S11. Tukey’s test for multiple comparisons at the 5% level of significance, comparing zones of inhibition produced by *Pseudomonas* isolate Roth107 in confrontation with different *Z. tritici* genotypes *in vitro*.

| Comparison | Mean Difference | Lower Bound | Upper Bound | Padj |
| --- | --- | --- | --- | --- |
| L951-IPO323 | -0.27970745 | -1.802888542 | 1.24347365 | 0.9999484 |
| Zt10-IPO323 | -0.76655142 | -2.289732514 | 0.75662968 | 0.8297251 |
| Zt114-IPO323 | 1.24400709 | -0.279174005 | 2.76718819 | 0.2020985 |
| Zt116-IPO323 | -1.09820035 | -2.62138145 | 0.42498074 | 0.3623116 |
| Zt118-IPO323 | 0.54451241 | -0.978668684 | 2.06769351 | 0.9806394 |
| Zt120-IPO323 | 0.25153369 | -1.271647407 | 1.77471478 | 0.9999822 |
| Zt36-IPO323 | -1.18480496 | -2.70798606 | 0.33837613 | 0.2597812 |
| Zt55-IPO323 | 0.69613918 | -0.827041911 | 2.21932028 | 0.8997142 |
| Zt74-IPO323 | 0.09932181 | -1.423859286 | 1.62250291 | 1 |
| Zt80-IPO323 | -0.25952571 | -1.782706805 | 1.26365539 | 0.9999756 |
| Zt92-IPO323 | 0.31906472 | -1.204116379 | 1.84224581 | 0.9998132 |
| Zt10-L951 | -0.48684397 | -2.010025068 | 1.03633712 | 0.99189 |
| Zt114-L951 | 1.52371454 | 0.000533441 | 3.04689563 | 0.0498527 |
| Zt116-L951 | -0.81849291 | -2.341674005 | 0.70468819 | 0.7657594 |
| Zt118-L951 | 0.82421986 | -0.698961238 | 2.34740095 | 0.7581682 |
| Zt120-L951 | 0.53124113 | -0.991939962 | 2.05442223 | 0.983925 |
| Zt36-L951 | -0.90509752 | -2.428278614 | 0.61808358 | 0.6428864 |
| Zt55-L951 | 0.97584663 | -0.547334466 | 2.49902773 | 0.5362282 |
| Zt74-L951 | 0.37902926 | -1.144151841 | 1.90221035 | 0.9990618 |
| Zt80-L951 | 0.02018174 | -1.50299936 | 1.54336283 | 1 |
| Zt92-L951 | 0.59877216 | -0.924408933 | 2.12195326 | 0.9616884 |
| Zt114-Zt10 | 2.01055851 | 0.487377413 | 3.53373961 | 0.0025229 |
| Zt116-Zt10 | -0.33164894 | -1.854830033 | 1.19153216 | 0.9997296 |
| Zt118-Zt10 | 1.31106383 | -0.212117266 | 2.83424493 | 0.1488991 |
| Zt120-Zt10 | 1.01808511 | -0.50509599 | 2.5412662 | 0.4734858 |
| Zt36-Zt10 | -0.41825355 | -1.941434642 | 1.10492755 | 0.9977402 |
| Zt55-Zt10 | 1.4626906 | -0.060490494 | 2.9858717 | 0.0694378 |
| Zt74-Zt10 | 0.86587323 | -0.657307869 | 2.38905432 | 0.7003882 |
| Zt80-Zt10 | 0.50702571 | -1.016155388 | 2.0302068 | 0.9887936 |
| Zt92-Zt10 | 1.08561613 | -0.437564962 | 2.60879723 | 0.3788678 |
| Zt116-Zt114 | -2.34220745 | -3.865388542 | -0.81902635 | 0.0002711 |
| Zt118-Zt114 | -0.69949468 | -2.222675775 | 0.82368642 | 0.8968586 |
| Zt120-Zt114 | -0.9924734 | -2.515654499 | 0.53070769 | 0.511317 |
| Zt36-Zt114 | -2.42881206 | -3.951993151 | -0.90563096 | 0.0001496 |
| Zt55-Zt114 | -0.54786791 | -2.071049003 | 0.97531319 | 0.9797327 |
| Zt74-Zt114 | -1.14468528 | -2.667866378 | 0.37849581 | 0.304686 |
| Zt80-Zt114 | -1.5035328 | -3.026713897 | 0.0196483 | 0.0557038 |
| Zt92-Zt114 | -0.92494237 | -2.448123471 | 0.59823872 | 0.6131166 |
| Zt118-Zt116 | 1.64271277 | 0.119531671 | 3.16589386 | 0.0252676 |
| Zt120-Zt116 | 1.34973404 | -0.173447053 | 2.87291514 | 0.1236805 |
| Zt36-Zt116 | -0.08660461 | -1.609785705 | 1.43657649 | 1 |
| Zt55-Zt116 | 1.79433954 | 0.271158443 | 3.31752064 | 0.0100835 |
| Zt74-Zt116 | 1.19752216 | -0.325658932 | 2.72070326 | 0.2465233 |
| Zt80-Zt116 | 0.83867465 | -0.684506451 | 2.36185574 | 0.7385999 |
| Zt92-Zt116 | 1.41726507 | -0.105916025 | 2.94044617 | 0.0881062 |
| Zt120-Zt118 | -0.29297872 | -1.81615982 | 1.23020237 | 0.9999185 |
| Zt36-Zt118 | -1.72931738 | -3.252498472 | -0.20613628 | 0.0150473 |
| Zt55-Zt118 | 0.15162677 | -1.371554324 | 1.67480787 | 0.9999999 |
| Zt74-Zt118 | -0.4451906 | -1.968371699 | 1.07799049 | 0.9961317 |
| Zt80-Zt118 | -0.80403812 | -2.327219218 | 0.71914297 | 0.7844774 |
| Zt92-Zt118 | -0.2254477 | -1.748628791 | 1.2977334 | 0.9999942 |
| Zt36-Zt120 | -1.43633865 | -2.959519748 | 0.08684244 | 0.0797977 |
| Zt55-Zt120 | 0.4446055 | -1.0785756 | 1.96778659 | 0.9961746 |
| Zt74-Zt120 | -0.15221188 | -1.675392975 | 1.37096922 | 0.9999999 |
| Zt80-Zt120 | -0.5110594 | -2.034240494 | 1.0121217 | 0.9880756 |
| Zt92-Zt120 | 0.06753103 | -1.455650068 | 1.59071212 | 1 |
| Zt55-Zt36 | 1.88094415 | 0.357763052 | 3.40412524 | 0.0058415 |
| Zt74-Zt36 | 1.28412677 | -0.239054323 | 2.80730787 | 0.1687723 |
| Zt80-Zt36 | 0.92527925 | -0.597901842 | 2.44846035 | 0.6126089 |
| Zt92-Zt36 | 1.50386968 | -0.019311416 | 3.02705078 | 0.0556013 |
| Zt74-Zt55 | -0.59681737 | -2.119998471 | 0.92636372 | 0.9625435 |
| Zt80-Zt55 | -0.95566489 | -2.47884599 | 0.5675162 | 0.5666825 |
| Zt92-Zt55 | -0.37707447 | -1.900255564 | 1.14610663 | 0.999105 |
| Zt80-Zt74 | -0.35884752 | -1.882028615 | 1.16433358 | 0.9994327 |
| Zt92-Zt74 | 0.21974291 | -1.303438189 | 1.742924 | 0.9999955 |
| Zt92-Zt80 | 0.57859043 | -0.94459067 | 2.10177152 | 0.9698637 |

Table S12. One-way Analysis of Variance of the radius of zones of inhibition produced by *Pseudomonas* isolate Roth112 in confrontation with genetically diverse *Z. tritici* isolates in the blastospore antagonism assay *in vitro*.

|  | Df | Sum Sq | Mean Sq | *F* value | Pr(>*F*) |  |
| --- | --- | --- | --- | --- | --- | --- |
| *Z. tritici* genotype | 11 | 63.63 | 5.785 | 8.11 | 6.92E-07 | *** |
| Residuals | 36 | 25.68 | 0.713 |  |  |  |

Significance codes: 0 ‘***’ 0.001 ‘**’ 0.01 ‘*’ 0.05 ‘.’ 0.1 ‘ ’ 1

Table S13. Tukey’s test for multiple comparisons at the 5% level of significance, comparing zones of inhibition produced by *Pseudomonas* isolate Roth112 in confrontation with different *Z. tritici* genotypes *in vitro*.

| Comparison | Mean Difference | Lower Bound | Upper Bound | Padj |
| --- | --- | --- | --- | --- |
| L951-IPO323 | 0.663989363 | -1.4203954 | 2.7483741 | 0.9921064 |
| Zt10-IPO323 | -1.180443262 | -3.264828 | 0.9039415 | 0.7050454 |
| Zt114-IPO323 | 1.284955675 | -0.7994291 | 3.3693404 | 0.5919036 |
| Zt116-IPO323 | -1.653439715 | -3.7378245 | 0.4309451 | 0.2356942 |
| Zt118-IPO323 | 1.950270389 | -0.1341144 | 4.0346552 | 0.0845742 |
| Zt120-IPO323 | 0.671023937 | -1.4133608 | 2.7554087 | 0.9914071 |
| Zt36-IPO323 | -1.308847517 | -3.3932323 | 0.7755373 | 0.5654938 |
| Zt55-IPO323 | -1.004388298 | -3.0887731 | 1.0799965 | 0.8647011 |
| Zt74-IPO323 | -1.6191578 | -3.7035426 | 0.465227 | 0.2614746 |
| Zt80-IPO323 | -1.004104609 | -3.0884894 | 1.0802802 | 0.8649097 |
| Zt92-IPO323 | -0.415172872 | -2.4995576 | 1.6692119 | 0.9998853 |
| Zt10-L951 | -1.844432625 | -3.9288174 | 0.2399521 | 0.1248323 |
| Zt114-L951 | 0.620966312 | -1.4634185 | 2.7053511 | 0.9954551 |
| Zt116-L951 | -2.317429078 | -4.4018138 | -0.2330443 | 0.0186877 |
| Zt118-L951 | 1.286281026 | -0.7981037 | 3.3706658 | 0.5904391 |
| Zt120-L951 | 0.007034574 | -2.0773502 | 2.0914193 | 1 |
| Zt36-L951 | -1.97283688 | -4.0572217 | 0.1115479 | 0.0776012 |
| Zt55-L951 | -1.668377661 | -3.7527624 | 0.4160071 | 0.2250374 |
| Zt74-L951 | -2.283147163 | -4.3675319 | -0.1987624 | 0.0217046 |
| Zt80-L951 | -1.668093972 | -3.7524787 | 0.4162908 | 0.2252366 |
| Zt92-L951 | -1.079162235 | -3.163547 | 1.0052225 | 0.8037089 |
| Zt114-Zt10 | 2.465398937 | 0.3810142 | 4.5497837 | 0.0096369 |
| Zt116-Zt10 | -0.472996453 | -2.5573812 | 1.6113883 | 0.9996003 |
| Zt118-Zt10 | 3.130713651 | 1.0463289 | 5.2150984 | 0.0003931 |
| Zt120-Zt10 | 1.851467199 | -0.2329176 | 3.935852 | 0.1217361 |
| Zt36-Zt10 | -0.128404255 | -2.212789 | 1.9559805 | 1 |
| Zt55-Zt10 | 0.176054964 | -1.9083298 | 2.2604397 | 1 |
| Zt74-Zt10 | -0.438714539 | -2.5230993 | 1.6456702 | 0.9998044 |
| Zt80-Zt10 | 0.176338653 | -1.9080461 | 2.2607234 | 1 |
| Zt92-Zt10 | 0.76527039 | -1.3191144 | 2.8496552 | 0.9764222 |
| Zt116-Zt114 | -2.93839539 | -5.0227802 | -0.8540106 | 0.0010174 |
| Zt118-Zt114 | 0.665314714 | -1.4190701 | 2.7496995 | 0.9919783 |
| Zt120-Zt114 | -0.613931738 | -2.6983165 | 1.470453 | 0.9958709 |
| Zt36-Zt114 | -2.593803192 | -4.678188 | -0.5094184 | 0.0053245 |
| Zt55-Zt114 | -2.289343972 | -4.3737287 | -0.2049592 | 0.0211277 |
| Zt74-Zt114 | -2.904113475 | -4.9884982 | -0.8197287 | 0.0012034 |
| Zt80-Zt114 | -2.289060284 | -4.3734451 | -0.2046755 | 0.0211538 |
| Zt92-Zt114 | -1.700128547 | -3.7845133 | 0.3842562 | 0.2035446 |
| Zt118-Zt116 | 3.603710104 | 1.5193253 | 5.6880949 | 0.0000364 |
| Zt120-Zt116 | 2.324463652 | 0.2400789 | 4.4088484 | 0.0181192 |
| Zt36-Zt116 | 0.344592198 | -1.7397926 | 2.428977 | 0.999982 |
| Zt55-Zt116 | 0.649051418 | -1.4353334 | 2.7334362 | 0.9934405 |
| Zt74-Zt116 | 0.034281915 | -2.0501029 | 2.1186667 | 1 |
| Zt80-Zt116 | 0.649335106 | -1.4350497 | 2.7337199 | 0.993417 |
| Zt92-Zt116 | 1.238266843 | -0.8461179 | 3.3226516 | 0.6432205 |
| Zt120-Zt118 | -1.279246452 | -3.3636312 | 0.8051383 | 0.59821 |
| Zt36-Zt118 | -3.259117906 | -5.3435027 | -1.1747331 | 0.0002069 |
| Zt55-Zt118 | -2.954658687 | -5.0390435 | -0.8702739 | 0.0009394 |
| Zt74-Zt118 | -3.569428189 | -5.653813 | -1.4850434 | 0.0000433 |
| Zt80-Zt118 | -2.954374998 | -5.0387598 | -0.8699902 | 0.0009407 |
| Zt92-Zt118 | -2.365443261 | -4.449828 | -0.2810585 | 0.0151161 |
| Zt36-Zt120 | -1.979871454 | -4.0642562 | 0.1045133 | 0.0755318 |
| Zt55-Zt120 | -1.675412234 | -3.759797 | 0.4089725 | 0.22014 |
| Zt74-Zt120 | -2.290181737 | -4.3745665 | -0.205797 | 0.0210509 |
| Zt80-Zt120 | -1.675128546 | -3.7595133 | 0.4092562 | 0.220336 |
| Zt92-Zt120 | -1.086196809 | -3.1705816 | 0.998188 | 0.7973926 |
| Zt55-Zt36 | 0.30445922 | -1.7799256 | 2.388844 | 0.9999949 |
| Zt74-Zt36 | -0.310310283 | -2.3946951 | 1.7740745 | 0.9999938 |
| Zt80-Zt36 | 0.304742908 | -1.7796419 | 2.3891277 | 0.9999949 |
| Zt92-Zt36 | 0.893674645 | -1.1907101 | 2.9780594 | 0.9320226 |
| Zt74-Zt55 | -0.614769503 | -2.6991543 | 1.4696153 | 0.9958231 |
| Zt80-Zt55 | 0.000283689 | -2.0841011 | 2.0846685 | 1 |
| Zt92-Zt55 | 0.589215426 | -1.4951693 | 2.6736002 | 0.9970924 |
| Zt80-Zt74 | 0.615053192 | -1.4693316 | 2.699438 | 0.9958068 |
| Zt92-Zt74 | 1.203984928 | -0.8803998 | 3.2883697 | 0.6802073 |
| Zt92-Zt80 | 0.588931737 | -1.495453 | 2.6733165 | 0.9971044 |
